# Mussel memory: can bivalves learn to fear parasites?

**DOI:** 10.1098/rsos.211774

**Published:** 2022-01-26

**Authors:** Christian Selbach, Loïc Marchant, Kim N. Mouritsen

**Affiliations:** ^1^ Department of Biology, Aquatic Biology, Aarhus University, Aarhus, Denmark; ^2^ Centre for Water and Environmental Research, University of Duisburg-Essen, Essen, Germany

**Keywords:** ecology of fear, non-consumptive effects, trematode, *Mytilus edulis*, *Himasthla elongata*

## Abstract

Fear plays a crucial role in predator–prey interactions and can have cascading impacts on the structure of whole ecosystems. Comparable fear effects have recently been described for hosts and their parasites but our understanding of the underlying mechanisms remains limited by the lack of empirical examples. Here, we experimentally tested if bivalves *Mytilus edulis* can potentially ‘learn to fear’ the infective transmission stages (cercariae) of the trematode *Himasthla elongata*, and if experienced mussels change their parasite-avoidance behaviour accordingly. Our results show that previous experience with parasites, but not established infections, lead to a reduced filtration activity in mussels in the presence of cercariae compared to parasite-naive conspecifics. This reduction in filtration activity resulted in lower infection rates in mussels. Since parasite avoidance comes at the cost of lower feeding rates, mussels likely benefit from the ability to adjust their defence behaviour when infection risks are high. Overall, these dynamic processes of avoidance behaviour can be expected to play a significant role in regulating the bivalves' ecosystem engineering function in coastal habitats.

## 1. Introduction

Fear saves lives, and fear shapes ecosystems. Fear can be defined as a central state that is induced when an organism perceives a threat or danger and triggers physical or behavioural responses [1,2]. Although such behavioural or physical responses are energetically costly, they increase fitness and have emerged as evolutionary stable strategies in different taxa [3]. For example, a herd of elk maintains constant vigilance against wolves or a school of fish moves in complex collective manoeuvres to confuse and avoid potential predators [4,5]. Such fear-induced predator avoidance behaviours can lead to trait-meditated indirect effects on other organisms in a community, and can have cascading impacts on the structure of whole ecosystems, comparable to or even exceeding the direct effects of predation [6–11]. Predator avoidance behaviour of herbivores (antelopes) in the African savannah, for instance, has been shown to shape the distribution and structure of Acacia tree communities in these habitats [12]. These ecological consequences of predator avoidance are referred to as the ‘ecology of fear' [3,13].

Similar to predators, parasites can induce ‘fear’ or ‘disgust’ in their hosts [14,15]. Free-living organisms have developed a variety of defence and avoidance strategies and mechanisms against parasites or pathogens, ranging from evading parasite transmission stages to avoiding interaction with infected conspecifics or staying clear of risky infection hot-spots, such as areas contaminated with faeces [14–18]. The ecological impacts of this fear of parasites have been shown to be comparable to the cascading effects of predator avoidance [19]. For instance, larval amphibians increase their activity to escape infective trematode cercariae in the water. This in turn not only impairs the tadpoles' grazing activity and growth rates, but also their susceptibility to predation, and the resource availability for other grazers, thereby affecting the food web dynamics in the ecosystem [14,20].

Overall, the capacity to avoid risks from predators or parasites requires that organisms have an intrinsic ability and/or can learn to differentiate between dangerous and safe conditions [4]. So far, most of the evidence that host organisms can learn to fear and avoid their parasites comes from terrestrial insects or mammals, while in aquatic systems, risk learning has been predominately studied for predator–prey interactions ([17] and reference therein). The most detailed and in-depth explorations of learning and parasite avoidance in aquatic environments come from trematode-fish interactions (e.g. [21,22]). For most other host–parasite systems, our understanding of these fundamental interspecific interactions in aquatic habitats remains limited by the lack of empirical examples. Changes in parasite avoidance behaviour as a result of experience and learning can have significant implications for disease dynamics and central ecological functions and need to be explored for further ecologically important host-parasite systems in aquatic environments [17,23].

Here, we use the bivalve *Mytilus edulis* and its trematode parasite *Himasthla elongata* as a model system to study the capability of hosts to potentially learn to avoid parasites and modify avoidance behaviour based on previous experience and assess the ecological implications of these processes. Blue mussels *M. edulis* are an important keystone species in Atlantic intertidal communities. They form extensive mussel beds along the coastline where they fulfil central ecological roles, such as filtering out organic matter and creating biogenic reefs, on which other organisms depend for shelter, substrate and foraging [24–27]. The trematode *H. elongata* uses blue mussels as a second intermediate host. Mussels become infected via free-swimming parasite dispersal stages, the cercariae, which are emitted from the common periwinkle snail *Littorina littorea* [28]. Once inside the mussel, the parasites encyst as metacercariae and can reduce growth rates [29], and render the mussel more vulnerable to predation by the parasite's final bird host [30]. Recent findings have shown that blue mussels try to avoid parasite infections by rapidly contracting their siphons and closing their shells when sensing cercariae in the water column [23]. However, this parasite avoidance strategy comes at a cost to the mussels, as it prevents them from filtrating and feeding during the parasite-avoidance phase [31].

In a set of microcosm experiments, we tested if mussels can learn to fear infective parasite stages in the water and if this process can modulate behavioural changes in their filtration activity in the presence of parasites. We hypothesized that mussels with previous parasite encounters would be more sensitive to this threat and exhibit stronger avoidance behaviours (shell closure and reduced filtration activity) compared to naive conspecifics. Overall, this could have important ecological implications, e.g. as it would impact the susceptibility of naive versus experienced mussels to parasite infections and thereby affect the distribution of parasites in an ecosystem. Moreover, this could have important implications for the ecosystem engineering potential of blue mussels, as mussels that gradually learn to avoid parasites by shutting down their filtration activity would remove less organic matter from the environment when parasites are present.

## 2. Material and methods

### 2.1. Collection of samples

Blue mussels *M. edulis* were provided by the Danish Shellfish Centre, Mors, Denmark. These mussels come from deeper sublittoral waters of the Limfjord, Denmark (56°53'29.2″ N 9°09'58.0″ E) where no *L. littorea* occur, ensuring that no mussels were infected by *H. elongata* [32]. A subsample of mussels was dissected to confirm the absence of trematode infections. All mussels were brought to the Marine Biological Station, Rønbjerg harbour, Limfjorden, Denmark, where all further treatment and experimentation was carried out. To obtain the parasites for infection experiments, periwinkle snails *L. littorea* were collected at an intertidal zone near Knebel in eastern Jutland, Denmark (56°12'32.2″ N 10°28'47.2″ E). Snails were screened for patent infections with *H. elongata*, and infected snails were isolated and kept dry in a climate chamber at 16°C until the start of the experiment.

### 2.2. Pre-experimental infection of mussels

To obtain infected mussels for the treatment groups (pre-infected group, see below), we experimentally infected blue mussels with freshly emitted cercariae of *H. elongata*. For this, 80 mussels were established in a 20 l aquarium with running sea water and air supply. Infected periwinkles were individually placed in glass jars with sea water at approximatively 25°C and placed under a light source to induce cercarial shedding. After 2 h, the glass jars were examined for released cercariae, and those containing free-swimming *H. elongata* were emptied into the aquarium containing mussels. At the same time, algae concentration (TETRASELMIS 3600, Instant Algae, Reed Mariculture) was added to the aquarium to induce filtration activity in mussels and increase infection success of the parasites. To allow a gradual build-up of trematode infections, mussels were repeatedly exposed to cercariae over the course of several days. The last batch of parasites was administered 24 h before the experiment. A second batch of mussels (*n* = 80) was treated the same way but received only filtered sea water and algae concentration without parasites (naive mussels, see below). Dissections of pre-infected mussels revealed that all individuals had acquired parasites during pre-experimental infections (ranging from 64 to 536 metacercariae, mean ± s.d. = 240 ± 144, *n* = 10).

### 2.3. Experimental design

To test if blue mussels can change their parasite-avoidance behaviour based on previous experience, we performed filtration experiments with pre-infected and naive mussels (i.e. without previous experience of parasites), either in the presence (‘exposed’ treatment) or absence (‘unexposed’) of trematode cercariae. Each treatment consisted of 10 replicates, resulting in a total of 40 samples. The experiment was carried out in a climate chamber at 16°C and performed in two batches of 20 mussels, five per treatment, over two days. To avoid confounding effects of mussel size, only individuals of similar size were used in the experiment (mean ± s.d. = 34.3 ± 1.5 mm, *n* = 40).

To obtain cercariae for the exposure treatments, freshly emitted *H. elongata* cercariae were collected in a Petri dish and separated in groups of 200 cercariae using a glass pipette. Each batch of cercariae was kept in 10 ml of sea water in a small Petri dish. Cercarial shedding and counting were done within 2 h before the start of the experiment, ensuring all cercariae were ≤2 h old and fully active.

Mussels were individually placed in a 1.5 l bucket with 1.1 l of filtered sea water and an air stone for oxygenation and allowed to acclimatize for 2 h before the start of the experiment. To measure filtration activity of the individual mussels, 0.4 l of water containing approximately 30 million cells of algae (TETRASELMIS 3600, Instant Algae, Reed Mariculture) was added to each bucket after the acclimation period, resulting in a final concentration of 20 million cells per litre. The exposure treatments each received 10 ml of sea water containing 200 *H. elongata* cercariae; the non-exposure treatments received the same amount of filtered sea water without cercariae.

After 2 h, all mussels were removed from the buckets and, after careful stirring, 0.5 l of water was removed from each bucket. Water samples were vacuum filtered through a 0.45 µm GC50 Glass Fibre Filter (Advantec, Japan) and filters were immediately frozen at −18°C for later chlorophyll-a analyses. Mussels were individually placed in a small bucket with filtered sea water for 12 h to allow metacercariae to establish before being frozen at −20°C until dissection.

### 2.4. Parasite infection intensity

Mussels were measured, dissected and the soft tissue tightly squeezed between two glass plates to quantify the infection intensity of *H. elongata*. Metacercariae were counted in the different mussel tissues under a stereomicroscope (ZEISS Stemi 2000c, Germany).

### 2.5. Measurements of chlorophyll-a concentration

Filters containing retained microalgae were transferred to dark test tubes together with 5 ml 96% ethanol and stored dark for 18 h at room temperature. The samples were then placed in an ultrasonic cleaner for 5 min followed by 20 s on a minishaker and finally 10 min centrifugation (4.000 r.p.m.). The resulting supernatant of each sample (3 ml) was transferred to a spectrophotometer (Spectronic Helios Alpha) and the absorbance was measured at 750 and 665 nm. Subsequently, the chlorophyll-a concentration (μg l^−1^) was calculated according to Riemann [33].

### 2.6. Data analyses

Statistical analyses were carried out in Statistical Package for the Social Science (IBM SPSS 27.0). All analyses were preceded by tests of homogeneity of error variance (Levene's test) and evaluation of normality. Three replica (i.e. individual mussels) were excluded in the final analyses: two mussels spawned heavily during the experiment, potentially affecting their filtration activity, and one mussel was excluded due to unreliable spectrophotometric absorbance measurement (at 750 nm). These omissions resulted in effective sample sizes of 9−10 per treatment.

Preliminary full model three-way ANOVA, entering chlorophyll-a concentration as dependent variable and parasite-exposure (absence/presence of cercariae), pre-infection status (pre-infected/naive) and experimental day as fixed factors, demonstrated neither day-interaction nor overall day-effect (*F*_1,29_ ≤ 0.758, *p* ≥ 0.391). Hence, data from the two experimental days were pooled in subsequent analyses in order to optimize statistical power. To test for differences between treatments, a full factorial two-way ANOVA was performed with post-experimental chlorophyll-a concentrations as dependent variable and parasite-exposure and pre-infection status as fixed factors. A linear regression was used to analyse the relationship between post-experimental total metacercarial load in mussels (log-transformed no. ind.^−1^) and chlorophyll-a concentration (μg l^−1^). For this, only parasite-naive mussels that were exposed to cercariae during the experiment were used, since all infections encountered in these individuals had to be acquired during the filtration experiment. All raw data including the infection intensities in mussels are provided in the supplemental material.

## 3. Results

**Figure 1.**
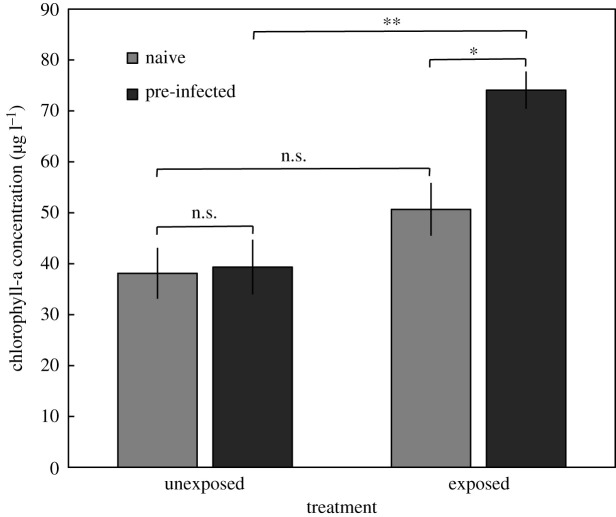
Post-experimental chlorophyll-a concentration (mean μg l^−1^ ± s.e.) after 2 h of filtration by blue mussels *Mytilus edulis* exposed and unexposed to *Himasthla elongata* cercariae. Naive: pre-experimentally uninfected mussels; pre-infected: mussels exposed to *H. elongata* prior to the experiment. *N* = 9–10 for each treatment combination. table 1 for summary statistics (main analysis). Asterisks above plots denote statistically significant differences between treatments (*p < 0.01; **p < 0.001; *n.s.* = not significant; Tukey HSD *post hoc* tests).

The two-way ANOVA, entering chlorophyll-a concentrations as dependent variable and experimental parasite-exposure and pre-infection status as fixed factors, showed significant two-way interaction as well as main effects (table 1 and figure 1). Overall, parasite exposure during the experiment caused a substantial reduction in the mussels' filtration activity (i.e. high chlorophyll-a concentration post-experimentally) explaining 41.5% of the variance (table 1). This effect was particularly pronounced among pre-infected mussels, which left a 46% higher chlorophyll-a concentration than parasite-naive mussels (figure 1). Already established infections had no bearing on the mussels’ filtration, as evident from the nearly identical chlorophyll-a concentrations for unexposed parasite naive and unexposed pre-infected mussels (figure 1). For the group of parasite-naive mussels, those exposed to *Himasthla* cercariae during the experiment left a 33% higher chlorophyll-a concentration than unexposed individuals. This difference was non-significant though (figure 1).

**Table 1.** Summary statistics of full model two-way ANOVA including post-experimental chlorophyll-a concentration (μg l^−1^) as dependent variable and parasite exposure (presence/absence of *Himasthla elongata* cercariae) and pre-infection status (pre-infected/parasite-naive) as fixed factors. Partial *η*^2^ denotes effect size, i.e. the proportion of variance explained.

source	d.f.	*F*	*p*	partial *η*^2^
pre-infection status	1	6.338	0.017	0.161
parasite exposure	1	23.365	<0.0005	0.415
interaction	1	5.139	0.030	0.135
error	33			

**Figure 2.**
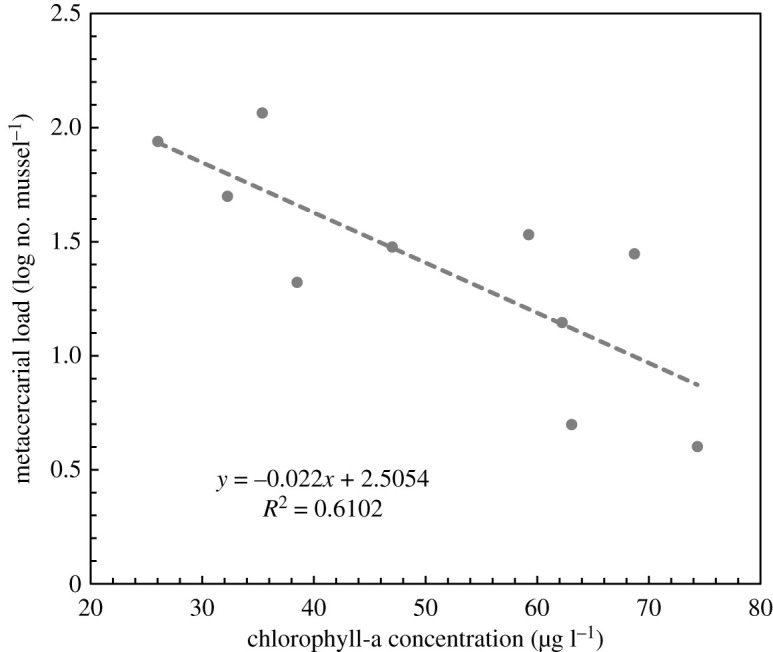
Relationship between post-experimental total metacercarial load in blue mussels *Mytilus edulis* (log-transformed no. ind.^−1^) and chlorophyll-a concentration (μg l^−1^) for pre-experimentally uninfected (naive) mussels exposed to *Himasthla elongata* cercariae. Linear regression: $r_{8}^{2}=0.610$, *p* = 0.008).

Focusing on parasite-naive mussels experimentally exposed to infective *Himasthla* larvae, there was a significant negative linear relationship between the post-experimental parasite load (log-transformed) and chlorophyll-a concentration (figure 2). This demonstrates that the rate by which parasite-exposed mussels acquired infections increases exponentially with filtration activity.

## 4. Discussion

Associative learning and risk avoidance have been shown for a range of predator–prey interactions but have received considerably less interest for host–parasite systems [22]. Accordingly, our understanding of host learning in parasite avoidance remains limited by the lack of empirical examples. In this study, we tested if blue mussels *M. edulis* can potentially ‘learn to fear’ the infective cercariae of the trematode parasite *H. elongata* in the environment and if experienced mussels change their parasite-avoidance behaviour accordingly. Our results show that previous parasite encounters, but not established trematode infections in the host, lead to a reduced filtration activity in mussels in the presence of cercariae compared to parasite-naive conspecifics. This confirms our hypothesis that bivalves adjust their behaviour depending on previous experience with the infective parasite stages to avoid additional infections. However, our results do not allow us to identify whether this behavioural change is due to associative learning based on specific clues, non-associative learning (sensitization, see [34]), or a state-dependent shift in parasite avoidance. While our findings support the hypothesis that mussels can ‘learn to fear’ their parasites, further testing will be required to examine the possible cognitive mechanisms behind these observed patterns. Regardless of the exact mechanisms, the reduced infection rates in mussels that showed lower filtration activities demonstrate the effectiveness of this avoidance reaction. The absence of any significant difference in chlorophyll concentration between pre-infected and naive mussels in the unexposed treatment shows that established infections of *H. elongata*, which predominantly encyst in the mantle and foot tissues [30], do not directly impact the mussels' filtration ability. Non-consumptive effects of *H. elongata* on *M. edulis* and the reduction of the mussels’ filtration activity as a result of parasite-induced avoidance behaviour have, recently, been discussed [23,31]. The results of our study now highlight the dynamic nature and plasticity of this interaction.

A parasite avoidance strategy that comes at the price of reduced filtration activity and feeding time can be considered highly costly for mussels [35]. For instance, reduced energy uptake due to defensive anti-parasite responses will likely result in lower growth rates and higher risks of predation, since smaller bivalves are more accessible to a wider range of predators [36,37]. A similar trade-off between parasite avoidance and other threats (predation and competition) could be demonstrated for amphibian tadpoles [14,38]. Overall, such parasite-defence strategies remain beneficial when the cost of infection exceeds the cost of avoiding parasites [39], but organisms should benefit from the ability to modulate their risk avoidance behaviour in relation to the actual threat level [17]. Blue mussels clearly show such an ability to adapt their risk behaviour depending on previous experience with trematodes, and can upregulate their avoidance behaviour when parasite pressure is high. It is unclear, however, if *M. edulis* will lose or ‘unlearn’ the fear of parasites again after prolonged periods of no contact with infective parasite stages. Infections of *H. elongata* in their first intermediate snail host and the release of cercariae into the environment typically peak during the warm summer months and recede in winter [40]. It is, therefore, feasible that mussels experience cyclical avoidance learning or sensitization phases each summer, followed by ‘forgetting’ or de-sensitization periods during the winter.

Moreover, recent studies have suggested that fish (sea trout *Salmo trutta trutta*) can change their defence strategies against trematodes (*Diplostomum pseudospathaceum*) over time and shift from avoidance learning to immunological defences after repeated exposure [22]. The present study can only present a snapshot in the temporal dynamics of parasite trait responses, which encompasses pre-contact, contact and post-contact phases of host–parasite interaction [18]. It, therefore, remains to be tested if bivalves can show a similar long-term plasticity in their defence against parasites, beyond dynamic and flexible avoidance behaviour (see [35]). Even more than prey, who often have several encounters with predators and learn to adjust their behaviour accordingly [4], blue mussels will have regular contact with trematode cercariae in their environment that will elicit defensive trait responses. It has been suggested that hosts generally have a broader and more diverse toolkit for defending against parasites due to this continuous interaction than prey have for defending against predators [18], which should be true for the *Mytilus*–trematode system as well.

As an ecosystem engineer that provides relevant ecological functions in coastal habitats, the parasite-mediated lower filtration rates of blue mussels are expected to influence the energy flow in coastal systems [23]. Based on the small-scale experiments in our study, it remains difficult to quantify how and to what extent the observed patterns of avoidance behaviour will affect blue mussels at the community and population level, and their overall ecosystem engineering potential, in particular the ability to remove organic matter from the water column. However, parasite avoidance behaviour might well have community-wide impacts similar to non-consumptive predator effects on the role of mussels as foundation species [11]. Overall, the avoidance patterns of mussels in regions with a high abundance of infected snails and high numbers of cercariae in the water, e.g. in shallow waters with high shorebird and periwinkle abundances [41], should be expected to be different from mussel populations facing only low parasite pressure, for instance in deeper waters where periwinkles are less common (such as the mussels used in this experiment). Based on our findings, it could be expected that mussels regularly facing trematode larvae in the environment become more risk averse than conspecifics in deeper waters. After regular parasite encounters, these populations could show different risk behaviours. Furthermore, within a community of mussels, not all individuals would be expected to show similar levels of avoidance behaviour based on varying experience levels and differences in personality, with less risk-averse mussels being more prone to infections [35,42]. This could be a contributing factor to the often uneven and patchy distribution of trematode infections in mussel populations [43].

Naturally, *H. elongata* is not the only threat blue mussels are regularly facing in their aquatic environment. Another trematode species, *Renicola roscovita*, also uses *Mytilus* as an intermediate host but encysts in gills and palps, where it directly impairs the mussels' filtration ability [44]. In intertidal mussel beds, both trematode species occur in sympatry and often infect the same mussels [40]. Since cercariae of both trematodes infect their bivalve host via similar infection pathways, learning to defend against one species might help to avoid another, thereby indirectly shaping parasite transmission dynamics of multiple species. Besides the risks of parasitism, a wide range of predators (birds, crabs, sea stars) prey on *M. edulis* in the intertidal zone. Bivalves have been shown to be capable of sensing and responding to risk cues from predators or alarm cues from conspecifics that face predation pressure [45–47]. Accordingly, future research should investigate potential additive or interactive fear effects of the simultaneous presence of parasites and predators in the context of avoidance learning [18].

Overall, non-consumptive and non-lethal effects of predators and parasites can have far-reaching cascading impacts on organisms in an ecosystem, and can even exceed consumptive effects. As we are increasingly uncovering how fear can shape ecosystems, the dynamic processes of avoidance behaviour that are likely playing an important role in these interspecific interactions and their ecological impact need to be studied in this context. Blue mussels and their trematode parasites provide an attractive and ecologically important model system to further explore these fundamental questions.

## Data accessibility

The datasets supporting this article have been uploaded as part of the electronic supplementary material [48].
